# Novel Targets and Interventions for Cognitive Complications of Diabetes

**DOI:** 10.3389/fphys.2021.815758

**Published:** 2022-01-04

**Authors:** Victoria Wolf, Yasir Abdul, Adviye Ergul

**Affiliations:** ^1^Ralph H. Johnson VA Medical Center, Charleston, SC, United States; ^2^Department of Pathology and Laboratory Medicine, Medical University of South Carolina, Charleston, SC, United States

**Keywords:** stroke, cognitive dysfunction, vascular dementia, intranasal, diabetes, VCID and intranasal treatment

## Abstract

Diabetes and cognitive dysfunction, ranging from mild cognitive impairment to dementia, often coexist in individuals over 65 years of age. Vascular contributions to cognitive impairment/dementia (VCID) are the second leading cause of dementias under the umbrella of Alzheimer’s disease and related dementias (ADRD). Over half of dementia patients have VCID either as a single pathology or a mixed dementia with AD. While the prevalence of type 2 diabetes in individuals with dementia can be as high as 39% and diabetes increases the risk of cerebrovascular disease and stroke, VCID remains to be one of the less understood and less studied complications of diabetes. We have identified cerebrovascular dysfunction and compromised endothelial integrity leading to decreased cerebral blood flow and iron deposition into the brain, respectively, as targets for intervention for the prevention of VCID in diabetes. This review will focus on targeted therapies that improve endothelial function or remove iron without systemic effects, such as agents delivered intranasally, that may result in actionable and disease-modifying novel treatments in the high-risk diabetic population.

## Introduction

The global incidence of diabetes has more than tripled in the last 20 years (International Diabetes Federation; IDF, 2019). Furthermore, the International Diabetes Federation with IDF estimates that there will be 578 million adults with diabetes by 2030, and 700 million by 2045 (IDF, 2019). This rise in the incidence of diabetes will continue to have devastating individual, social, and economic consequences resulting from high mortality and morbidity due to complications associated with the disease. While the ultimate goal is to prevent diabetes and find a cure for the disease, prevention and treatment of complications are equally important. Most if not all diabetic complications are associated with vascular disease. Traditionally, diabetic complications are classified as microvascular (nephropathy, retinopathy, and neuropathy) and macrovascular (heart disease, stroke, and peripheral arterial disease; Stettler et al., 2006). In recent years, there is increasing recognition that cognitive impairment, ranging from mild cognitive dysfunction to dementia, is another neurological complication of diabetes with vascular origins (Gorelick et al., 2017; van Sloten et al., 2020) as numerous studies have shown that decreased cerebral blood flow (CBF) and increased blood brain barrier (BBB) permeability precede the development of neuronal pathologies and overt cognitive deficits (Iadecola, 2017; Sweeney et al., 2019a). Unfortunately, there are no treatments for VCID. Studies including ACCORD-MIND and Veterans Affairs Diabetes Trial showed that glycemic control does not prevent cognitive decline, suggesting hyperglycemia is not the sole factor (Zimering et al., 2016; van Sloten et al., 2020). There is an urgent need to understand the underlying mechanisms linking diabetes to VCID in order to develop mechanism based effective therapies. In preclinical studies, we have identified cerebrovascular dysfunction and compromised neurovascular unit (NVU) as therapeutic targets and started testing multiple pharmacological interventions to correct these pathologies for the prevention and treatment of VCID in diabetes. In this series focusing on “Novel Targets and Drug Delivery Systems in Treating Diabetic Complications,” we will first review the microvascular basis of VCID in diabetes and then discuss the emerging field of intranasal (IN) drug delivery in neurodegenerative diseases. Finally, we will discuss how the IN use of the pharmacological interventions we identified can be applied to VCID associated with diabetes.

## Cerebrovascular Complications of Diabetes: Stroke and Cognitive Dysfunction

About 100 million people in the United States suffer from diabetes or pre-diabetes. As briefly discussed above, individuals with diabetes are at 2–4-times increased risk of cerebrovascular diseases like ischemic stroke and cognitive dysfunction, including vascular cognitive impairment (VCI) and post stroke cognitive impairment (PSCI), which collectively contribute to the VCID spectrum (Corriveau et al., 2016; Li et al., 2017; Lo et al., 2019, 2020; Shang et al., 2020; Virani et al., 2020). Ongoing research efforts are trying to clarify the independent and interacting risks and causes that lead to both type 2 diabetes and neurovascular diseases (Megherbi et al., 2003; Saczynski et al., 2008; Launer et al., 2011). Diabetes affects both small and large blood vessels that can lead to multiple end organ damage, including the nervous system. Although greater stroke occurrence in diabetes is mainly attributed to accelerated atherosclerosis of the macrovasculature, it is increasingly recognized that microvascular dysfunction contributes to stroke occurrence and PSCI (Biessels and Despa, 2018; Biessels et al., 2020; Biessels and Whitmer, 2020; van Sloten et al., 2020).

Regulation of cerebrovascular integrity and function is key in proper cerebral perfusion and neuronal function (Coucha et al., 2018). In this regard, the NVU concept offers a framework to interrogate the complex and dynamic interactions between vascular cells (endothelial cells, pericytes, and smooth muscle cells of penetrating arterioles), glial cells (astrocytes, microglia, and oligodendrocytes) and neurons (Iadecola, 2017; Sweeney et al., 2019b). Microvascular dysfunction can manifest as compromised cerebral autoregulation, cerebrovascular hyperreactivity, and/or loss of endothelial integrity leading to increased BBB permeability and loss of trophic support that glial and neuronal cells depend upon for survival (Fulesdi et al., 1997; van Elderen et al., 2011; Gorelick et al., 2017; Iadecola, 2017; van Sloten et al., 2020). Endothelial dysfunction and poor cerebral autoregulation can occur early in diabetes and in due time can lead to hypoperfusion (Coucha et al., 2018). The hypoxic microenvironment mediates pathological angiogenesis, which can worsen the existing BBB compromise, and triggers a feed forward inflammatory loop resulting in white matter abnormalities, microbleeds, and large and micro infarcts, as well as enlarged perivascular spaces (Sweeney et al., 2019b; van Sloten et al., 2020; Wardlaw et al., 2020b). A recent study proposed a mechanistic framework and discussed how cerebrovascular dysfunction in diabetes can potentially lead to a mild hypoxic state and exacerbate the metabolic dysfunction-driven suppression of neuronal autophagy leading to cognitive impairment (Fakih et al., 2020).

From a clinical perspective, increased hemoglobinA1C is associated with decreased CBF due to vasogenic edema and reduction in cerebral autoregulation (Last et al., 2007; McCormick et al., 2010). Microvascular dysfunction in type 2 diabetes has been reported to be associated with increased risk of ischemic stroke, especially lacunar strokes (Liu et al., 2018; Cukierman-Yaffe et al., 2021). Studies also showed increased BBB permeability in individuals with type 2 diabetes that was also associated with worse functional outcomes after stroke (Luitse et al., 2012; Palacio et al., 2014; Pan et al., 2016; Yu et al., 2016; van Sloten et al., 2020). An important consequence of cognitive dysfunction in diabetes is that the decline in executive function can complicate the management of diabetes in these individuals creating a vicious cycle. Evidence supporting the involvement of cerebral microvascular dysfunction in individuals with diabetes associated cognitive impairment was recently summarized in an elegant review (van Sloten et al., 2020).

From a preclinical perspective, experimental models of VCID and metabolic disease have lower CBF, greater white matter damage, reduced oligodendrocytes progenitor cells, impaired spatial memory, and reduced cue fear memory along with neurodegeneration (Kelly-Cobbs et al., 2012; Kwon et al., 2015; Yatomi et al., 2015; Zuloaga et al., 2016). It has also been shown that diabetes promotes excessive pathological neovascularization in the brain (Li et al., 2010; Prakash et al., 2012, 2013a,b). An ischemic injury layered on this pathology causes greater hemorrhagic transformation into the brain, which promotes vascular rarefaction and poor stroke recovery, including augmented cognitive deficits (Elgebaly et al., 2010; Li et al., 2013, 2017; Prakash et al., 2013b; Abdelsaid et al., 2015; Ward et al., 2018b).

Collectively, it is evident from both clinical and experimental studies that diabetes-mediated microvascular dysfunction plays a critical role in the development and progression of VCID by promoting BBB disruption, exacerbating neurovascular damage after ischemic injury and impairing vasotrophic coupling necessary for the repair processes. Innovative approaches to improve cerebral microvascular function, endothelial integrity, and trophic coupling within the NVU hold great potential to develop preventive and therapeutic strategies for diabetes-associated cognitive dysfunction. Thus, after a brief summary of IN drug delivery, we will review the potential IN use of insulin, iron chelator deferoxamine (DFX), phosphodiesterase-3 (PDE3) inhibitor cilostazol, endothelin (ET) receptor antagonists, and brain derived neurotrophic factor (BDNF) for improvement of microvascular function and vasoneuronal health in diabetes.

## Intranasal Drug Delivery

IN drug delivery is an emerging field in neurodegenerative diseases. Currently, potential treatments for ADRD, including VCID, are limited by the physiological and anatomical characteristics of the central nervous system (CNS; Cunha et al., 2021). IN delivery can also be used to repurpose drugs, which are already used to treat classical symptoms of diabetes, for the management of cerebrovascular complications of the disease (Jojo et al., 2019). IN delivery makes it possible to achieve a significantly higher concentration of a drug in the brain to induce a maximum therapeutic response. This is especially important when the drug being considered has dose-dependent peripheral side effects when administered systemically (Jojo et al., 2019). The BBB not only provides protection against xenobiotics but also limits entry of ~98% of low molecular weight and 100% of high molecular weight drugs (Erdő et al., 2018; Cunha et al., 2021). Hydrophilic substances, charged molecules, proteins, and peptides are unable to cross the BBB, but lipophilic drugs, such as antidepressants, anxiolytics, and many hormones can more easily cross (Khan et al., 2017). The passage of molecules through the endothelial cells and tight junctions of the BBB depends on their physiochemical characteristics and interaction with endogenous efflux transports, i.e., ATP binding cassette transporters (Cunha et al., 2021).

**Figure 1:**
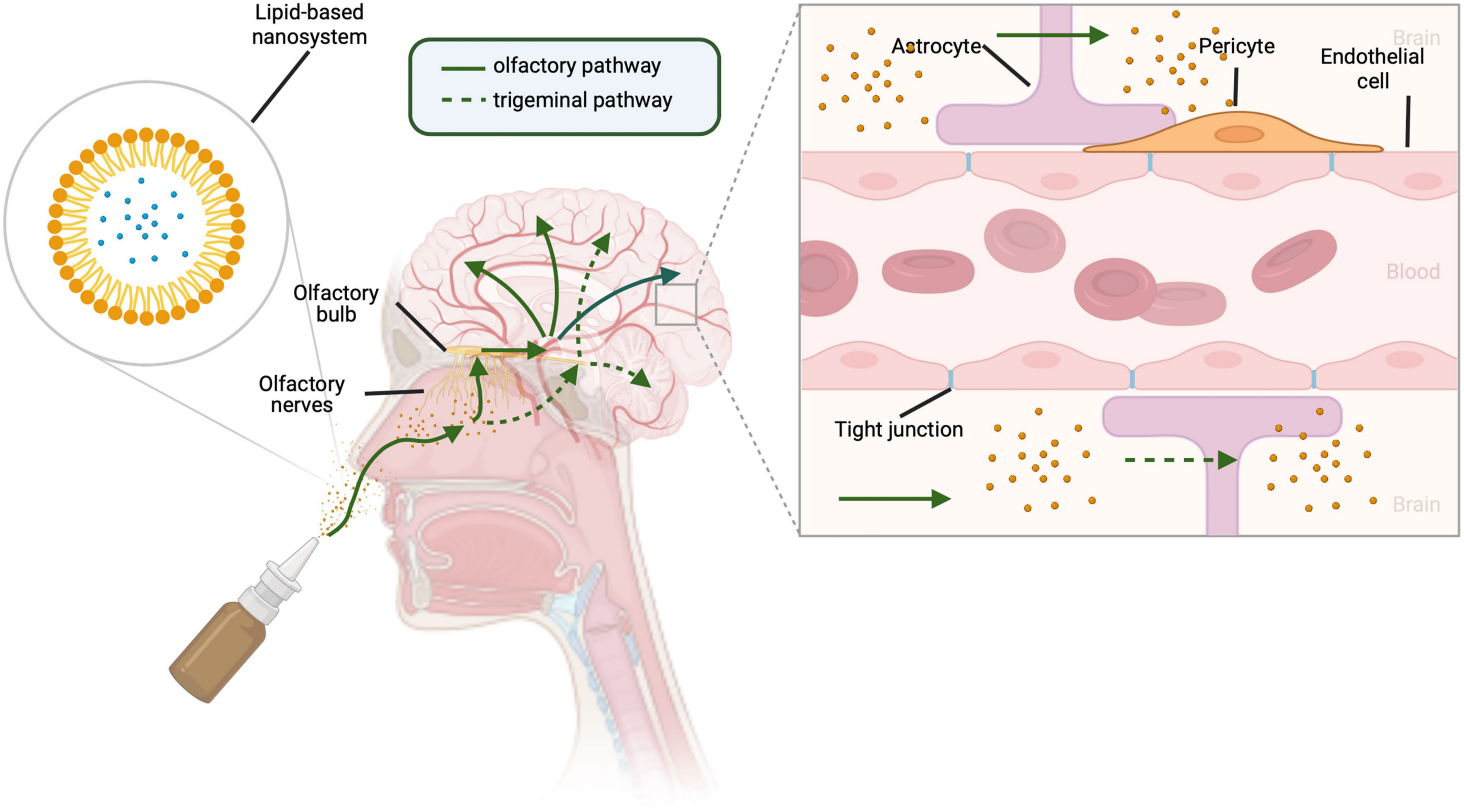
Routes for intranasal delivery of drugs to the central nervous system. Lipophilic drugs can be packaged in lipid-based carrier systems. Upon entering the nasal cavity, drugs can reach the brain by either olfactory (solid green lines) or trigeminal nerve (dotted green lines) pathways. Traversing branches of the trigeminal nerve, drugs enter the brain *via* the trigeminal ganglion and brainstem. Olfactory neurons send cilia into the nasal cavity lumen, a major site for drug absorption, providing access to the olfactory bulb and then to the brain. This allows drugs to bypass the endothelial cells, pericytes, astrocytes, and tight junctions that form the blood brain barrier. Created with BioRender.com.

IN delivery involves the direct delivery of therapeutics from the nasal cavity into the CNS, bypassing the BBB and providing a less invasive method of drug administration (Hanson and Frey, 2008). Figure 1 illustrates the routes of IN delivery that have been identified for access to the CNS. The field first emerged in 1989 when Frey developed an IN formulation for targeting neurotrophic factors to the CNS (Hanson and Frey, 2008). Since then, IN delivery to the CNS has been used for numerous other classes of therapeutics: neuropeptides, cytokines, polynucleotides, and small molecules (Hanson and Frey, 2008). Pharmaceuticals have been developed for various IN formulations, including powders, nasal sprays, *in situ* hydrogels, and nanosystems (Cunha et al., 2021). Nanosystem formulations are currently considered as the most promising strategy for IN delivery formulations targeting the brain (Jojo et al., 2019). Lipid-based nanosystems, such as nanoemulsions and nano-lipid carriers (NLCs), have been identified as efficient systems to deliver lipophilic drugs, providing protection from enzymes and mucociliary clearance in the nasal cavity (Cunha et al., 2021). Nanoemulsions have a mean droplet size of 20–200 nm and are composed of two immiscible phases (water and oil) that are stabilized by one or two emulsifiers (Cunha et al., 2021). NLCs have a high entrapment efficacy, stability, and property to accommodate both lipophilic and hydrophilic molecules, making it superior over other lipid nanocarriers (Jojo et al., 2019).

As illustrated in Figure 1, olfactory and trigeminal neural pathways both contribute to the IN route to the CNS (Hanson and Frey, 2008) and provide a beneficial pharmacokinetic/pharmacodynamic profile for CNS acting drugs (Erdő et al., 2018). The extensive vascularization of the mucosa, lamina propria, and the leaky epithelium provide an optimal absorption surface for drug delivery (Wengst and Reichl, 2010; Lungare et al., 2016). The transportation of molecules from the nasal cavity to the parenchyma of the brain occurs *via* intracellular and extracellular pathways. The intracellular pathway involves (1) internalization of the molecule by an olfactory neuron, (2) endocytic vesicular trafficking to the neuron’s projection site, and (3) release by exocytosis (Erdő et al., 2018). The extracellular pathway involves (1) the drug crossing the nasal epithelium to the lamina propria where neurons are located, especially in the olfactory region of the nasal cavity, (2) transportation along the length of the neuronal axon by bulk flow processes, and (3) further distribution *via* fluid movement into the CNS (Erdő et al., 2018). There is a short time frame (≤10 min) for delivery to the brain from the nasal mucosa, suggesting a predominantly extracellular mechanism of delivery, rather than axonal transport (Hanson and Frey, 2008).

Labeled macromolecules, such as interferon gamma, insulin-like growth factors, and insulin, have demonstrated rapid uptake into the brain and cerebral spinal fluid spaces along the trigeminal route following IN administration (Thorne et al., 2004, 2008; Lochhead et al., 2019). Limitations of IN delivery to the CNS include the relatively small volume for administration of the drugs, limited surface area of the olfactory epithelium, short retention time for drug absorption, and the influence of nasal secretions on drug delivery (Wu et al., 2008). To achieve the highest probability of transport, a drug needs to exhibit moderate hydrophilic properties to minimize hydrophobic interactions with the mucus and maintain the ability to be dissolved in aqueous medium, all while avoiding clearance (Gänger and Schindowski, 2018). Mucin is negatively charged, so to minimize electrostatic interactions that cause drug entrapment within the mucous, the drug needs to be neutral or slightly negative at physiological pH (Gänger and Schindowski, 2018). Surface modifications and thermosensitive and mucoadhesive polymers have been used to enhance the therapeutic potential of lipid-based nanosystems and reduce adverse effects (Cunha et al., 2021). These are important considerations for the development of new IN delivery formulations for emerging therapeutic targets for VCID in diabetes.

## Therapeutic Targets

### Insulin

Enhancement of insulin levels and/or insulin sensitivity is at the epicenter of diabetes management. Insulin, 5.8 kDa hormone composed of two peptide chains, is released from islet cells of the pancreas in response increased plasma glucose levels. Binding to its receptors in target organs (liver, muscle, and adipose tissue) promotes its well-studied metabolic effects such as increased glucose uptake/utilization *via* stimulation of glucose transporter type 4 (GLUT4) in muscle and adipose tissue, greater glycogen synthesis in liver and muscle, enhanced protein synthesis, and decreased lipolysis. Identification of insulin and its receptors in the brain around 1980s (Havrankova et al., 1978a,b) and the demonstration of the impact of cerebroventricular administration of insulin on food intake and body weight in baboons led to subsequent investigations into the CNS effects of insulin (Woods et al., 1979). The wide distribution of insulin receptors suggested complex roles of insulin in the CNS. Coupled with the further discovery that the transport of insulin into the brain is reduced with diabetes and in patients with cognitive dysfunction led to the recognition of brain as a new target organ for insulin (Heni et al., 2014; Stanley et al., 2016).

**Figure 2:**
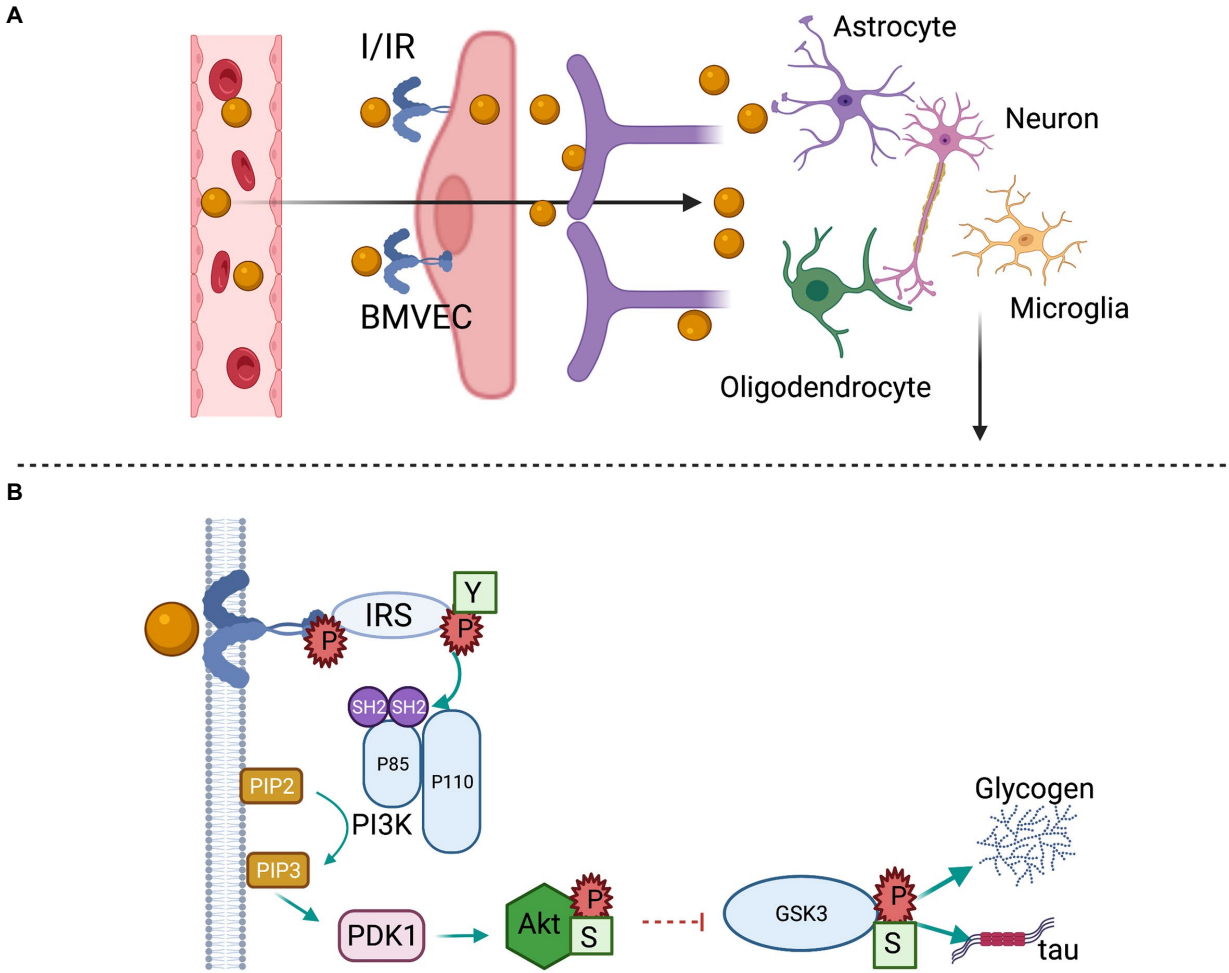
Brain insulin transport and signaling. **(A)** Circulating insulin (I) binds the insulin receptor (IR) on the luminal membrane of brain microvascular endothelial cells (BMVECs). After crossing the endothelial cell layer, insulin is released at the basement membrane and may interact with pericytes, astrocytes, or act directly on neurons. **(B)** The activated IR has tyrosine kinase activity that initiates a cell signaling cascade. Insulin receptor substrate (IRS) proteins are phosphorylated at tyrosine residues (Y), which bind to the Src homology 2 (SH2) domains of the p85α subunit of phosphoinositide-3 kinase (PI3K) and catalyze the formation of phosphatidylinositol-3,4,5-trisphosphate (PIP_3_). PIP_3_ catalyzes the formation of phosphoinositide-dependent kinase (PDK1), which phosphorylates and activates Akt at Serine (S) 473. The activated Akt phosphorylates and inhibits glycogen synthase kinase 3 (GSK3) β at Serine 9, stimulating glycogen synthesis and the phosphorylation of tau protein.

Insulin enters the brain parenchyma *via* a saturable transendothelial process by binding to insulin receptors on endothelial cells that form the BBB and then is released at the basal membrane (Gray and Barrett, 2018). A second route is through the delivery of cerebrospinal fluid (Figure 2A). In addition to the brain microvascular endothelial cells (BMVECs), neurons and glial cells including astrocytes, microglia, and oligodendrocytes have insulin receptors. As depicted in Figure 2B, binding of insulin to the extracellular α-subunits mediates dimerization and autophosphorylation of the intracellular β-subunits, followed by the tyrosine phosphorylation of insulin receptor substrate (IRS) proteins, especially IRS-1, with subsequent activation of phosphoinositide 3 kinase (PI3K) and downstream Akt phosphorylation. This leads to regulation of transcription factors, such as FOXO1, and subsequent transcription of genes involved in key functions in neurons and glia. In addition, Akt can inhibit glycogen synthase kinase 3β (GSK3β), a constitutively active enzyme that not only regulates glycogen synthase but also promotes microtubule-associated tau protein phosphorylation, which contributes to neurofibrillary tangle formation in neurodegenerative diseases (Erichsen et al., 2021; Scherer et al., 2021). The lack of these central effects of insulin, either due to poor transport into brain or impaired insulin signaling, lead to many metabolic abnormalities. We refer the readers to an excellent review for systemic metabolic consequences (Scherer et al., 2021), and we will focus on cognitive consequences and IN insulin use in the following paragraphs.

The seminal study by Stephen Woods and colleagues in which they demonstrated that chronic intracerebroventricular administration of insulin reduces food intake and body weight of baboons paved the way to preclinical and clinical studies with the IN insulin to enhance brain insulin delivery and signaling (Woods et al., 1979). It soon became clear that insulin also impacts cognitive functions leading to numerous studies in healthy volunteers as well as in individuals with ADRD over the last two decades (Avgerinos et al., 2018; Erichsen et al., 2021; Hallschmid, 2021a,b). A majority of these studies provided evidence that the IN insulin is safe and well tolerated while revealing a great potential for memory-enhancing and/or memory-preserving effects (Schmid et al., 2018). These studies also showed that several factors, including insulin formulation, length of treatment, sex, dose, and APOE genotype, affect the treatment response (Avgerinos et al., 2018; Erichsen et al., 2021; Hallschmid, 2021a,b). Preclinical studies in rodent models corroborated human studies. Although some studies showed IN insulin decreases anxiety-like behavior and improves various memory tasks, other studies were neutral, again pointing to the impact of dose, duration, genetic background, age of the animals, and insulin formation (Erichsen et al., 2021). Thus, these preclinical models not only provide an excellent platform to refine and improve IN insulin therapy but also to identify the underlying mechanisms contributing to CNS effects of insulin on cognitive functions.

### Iron Chelators

Iron is a crucial element for many fundamental and specialized processes in biology, including but not limited to cellular respiration, oxygen transport, enzymatic activity, neurotransmission, and myelin homeostasis (Masaldan et al., 2019; Kosyakovsky et al., 2021). The dynamic oxidation state between ferric Fe^3+^ and ferrous Fe^2+^ forms is essential for its biological actions, but it can also contribute to oxidative stress in disease conditions. As such, there are multiple mechanisms that regulate circulating and tissue free iron as briefly discussed below. Advances in the imaging modalities led to the recognition that focal iron accumulation in the brain is a common pathological finding in many neurodegenerative diseases including Parkinson’s disease, amyotrophic lateral sclerosis, and AD. In this review, we will focus on AD and related VCID and refer the readers to recent reviews for other neurodegenerative diseases (Devos et al., 2020; Farr and Xiong, 2021; Kosyakovsky et al., 2021).

Circulating iron is bound to transferrin and enters the brain by interacting with transmembrane transferrin 1 (TfR1) receptors and divalent metal transporter-1 (DMT-1) on BMVECs and to a lesser extent in epithelial cells of the choroid plexus (Moos et al., 2007; Simpson et al., 2015). Once in endothelial cells, iron is rapidly bound by ferritin and the excess is transported out of the cell toward the brain parenchyma by ferroportin. More detailed information on brain iron homeostasis can be found in an excellent review by Morris et al. (2018). Accumulation of iron in the brain in AD and VCID may be due to impaired BBB integrity that occurs early in the disease process before neuropathologies and cognitive deficits can be detected. Exacerbated BBB disruption in diabetes may further aggravate brain iron deposition. Indeed, recent imaging studies reported increased brain iron in patients with diabetes and deficits in executive function (Yang et al., 2018; Li et al., 2020, 2021). In the case of PSCI, diabetes increases hemorrhagic transformation, secondary bleeding into brain after ischemic stroke in patients and experimental models (Martini and Kent, 2007; Ergul et al., 2012), and this may lead to iron deposition in the brain.

While the exact mechanisms of iron contribuion to neurovascular pathologies are not fully understood, compounding evidence points to ferroptosis, an iron dependent form of cell death (Dixon et al., 2012; Magtanong and Dixon, 2018; Derry et al., 2020). In the above-mentioned neurodegenerative diseases, iron accumulation in the brain does not reach toxic levels. Nevertheless, due to its dynamic redox state, it can promote oxidative stress facilitating and accelerating ferroptosis, a nonapoptotic form of cell death that is differentiated by iron-dependent accumulation of reactive oxygen species, leading to lasting lipid damage and membrane permeabilization. Prevention of cell death by an iron chelator such as deferoxamine (DFX) is a prerequisite to identify ferroptosis (Magtanong and Dixon, 2018). Earlier studies by our group reported cerebral vasoregression that is associated with greater hemorrhagic transformation after stroke and accompanying PSCI in diabetic rats (Elgebaly et al., 2010; Li et al., 2013; Prakash et al., 2013b). Building upon these observations, a more recent study showed that systemic DFX treatment after stroke prevented PSCI, and this was associated with preserved cerebral vascularization in diabetes (Li et al., 2013; Abdul et al., 2021). In addition to the published studies, which reported iron-mediated ferroptosis in neurons (Zille et al., 2017), this study also provided evidence for ferroptosis in BMVECs, which may be responsible for stroke-mediated vasoregression. Collectively, these findings suggest that iron negatively affects the entire NVU and DFX can protect all components of the NVU.

Well documented evidence for iron accumulation in the brain in neurodegenerative diseases led to preclinical and clinical investigations with iron chelators. DFX, an FDA-approved iron chelator routinely used in iron overload cases, is the most commonly tested iron chelator in these studies (Devos et al., 2020; Farr and Xiong, 2021; Kosyakovsky et al., 2021). While it appears to have great potential to prevent iron accumulation, DFX also has several limitations. Due to its short half-life, it requires multiple injections. It also has side effects such as hearing loss, loss of vision, and growth retardation in children and these adverse effects are often dose related (Brittenham, 2011; Di Nicola et al., 2015; Bayanzay and Alzoebie, 2016). IN DFX may be an alternative to overcome these issues. A number of preclinical studies provided convincing evidence that IN DFX is effective in reducing plaque burden, decreasing tau hyperphosphorylation and improving memory and learning deficits in AD models (Fine et al., 2012, 2015, 2017; Hanson et al., 2012; Guo et al., 2013a,b, 2015). It was shown that IN DFX upregulates BDNF, a critical neurotrophic factor with angiogenic properties, that is also being tested for IN delivery as discussed below (Guo et al., 2015). Interestingly, one of these studies was conducted in intracerebroventricular streptozotocin injection model of sporadic AD characterized with irregular insulin metabolism (Fine et al., 2017). The impact of IN DFX and/or other iron chelators in diabetes-associated VCID remains to be determined.

### PDE3 Inhibitors

Cyclic adenosine monophosphate (cAMP or 3′,5′-cyclic adenosine monophosphate) and guanosine monophosphate (cGMP or 3′,5′-cyclic guanosine monophosphate) are important second messengers in signaling cascades that regulate many biological processes. cAMP generated by adenylate cyclase activates protein kinase A (PKA). cGMP generated by soluble guanylyl cyclase upon activation by nitric oxide (NO) activates PKG. Both cAMP and cGMP are rapidly hydrolyzed by various members of the phosphodiesterase (PDE) family of enzymes, most of which are expressed in the brain (Maki et al., 2014; Sharma et al., 2020). PDE3 can degrade both cyclic nucleotides. Inhibition of PDEs maintains cAMP and cGMP levels. In the CNS, cAMP/PKA and cGMP/PKG pathways lead to activation of cAMP response element binding protein (CREB), which leads to transcription of numerous genes critical for synaptogenesis, memory function, and mitochondrial biogenesis, such as activity-regulated cytoskeleton-associated protein (Arc), BDNF, and nuclear respiratory factor (Nrf1; Menniti et al., 2006; Kandel, 2012; Sanders and Rajagopal, 2020). These cyclic nucleotides are also critical for regulation of vasoreactivity (Maki et al., 2014; Sanders and Rajagopal, 2020). As discussed earlier, vascular dysfunction is an early finding in diabetes as well as in all forms of dementias. Thus, dysregulation of these signaling molecules can contribute to the pathogenesis and progression of diabetes-associated cognitive decline *via* multiple mechanisms.

Indeed, the potential role of PDE inhibitors for the treatment of AD has been investigated in various preclinical and clinical studies (Menniti et al., 2006; Prickaerts et al., 2017; Yanai and Endo, 2019; Sanders and Rajagopal, 2020). For the purposes of this review, we will focus on PDE3 inhibition because PDE3 has the highest affinity for both cAMP and cGMP (Yanai and Endo, 2019). We will also concentrate on cilostazol because of its antiplatelet, vasodilatory, neuroprotective, and anti-inflammatory effects, all of which are important for the pathophysiology of VCID (Maki et al., 2014; Yanai and Endo, 2019; McHutchison et al., 2020). Cilostazol is used for peripheral vascular disease in Western countries and for secondary stroke prevention in Asian countries. Cilostazol has been tested alone or in combination with other medications in numerous clinical trials for ischemic stroke, AD, and VCID as recently discussed in a review article (Prickaerts et al., 2017; Yanai and Endo, 2019) and a meta-analysis (McHutchison et al., 2020). Two ongoing trials include the LACI-2 (LACunar Intervention Trial-2) and the COMCID (The Cilostazol for prevention of COnversion from MCI to Dementia; Saito et al., 2016; Wardlaw et al., 2020a). LACI-1 trial showed tolerability and early markers of safety and efficacy in patients with lacunar stroke for cilostazol alone or in combination with isosorbide mononitrate (ISMN; Blair et al., 2018). For the purposes of this review, it is important to indicate that patients with diabetes suffer from lacunar strokes. All these studies suggested that cilostazol is a promising drug for secondary stroke prevention with low risk of hemorrhage due to its weak antiplatelet effects, and the data related the efficacy in prevention of cognitive decline in LACI-2 and COMCID trials are anxiously awaited.

Years of investigation in preclinical studies demonstrated that cilostazol prevents aberrant tau and Aβ deposition (Maki et al., 2014; Saito et al., 2016; Sanders and Rajagopal, 2020). An interesting study showed that cilostazol improves impaired cerebral autoregulation in the OLEFT rat model of diabetes *via* upregulation of endothelial nitric oxide synthase (eNOS) phosphorylation and vascular endothelial growth factor (VEGF) expression (Tsukamoto et al., 2017). Another study reported that diabetes worsens cognitive function in a hypoperfusion model of VCID, and this can be prevented by cilostazol treatment (Kwon et al., 2015). Diabetes has been recently shown to worsen neurovascular pathologies in a microemboli (ME) model of VCID (Chandran et al., 2020). Follow up imaging studies suggested that preventive treatment with cilostazol before ME injection prevented ME-mediated pathologies in the microstructure of gray and white matter (unpublished data).

These promising observations are hampered by poor solubility and low BBB penetrance (Saito et al., 2016; Yoshioka et al., 2018). An oral nanoparticle formulation has been shown to have better absorption. There is one publication in Chinese that reported IN cilostazol given for 2 weeks after stroke surgery improves neuronal survival in mice, but there is no data on cognitive outcomes (Zhang et al., 2011). Based on the clinical and experimental studies, we postulate that IN cilostazol may be a viable and practical option for the prevention and treatment of diabetes-associated VCID.

### Endothelin Receptors

**Figure 3:**
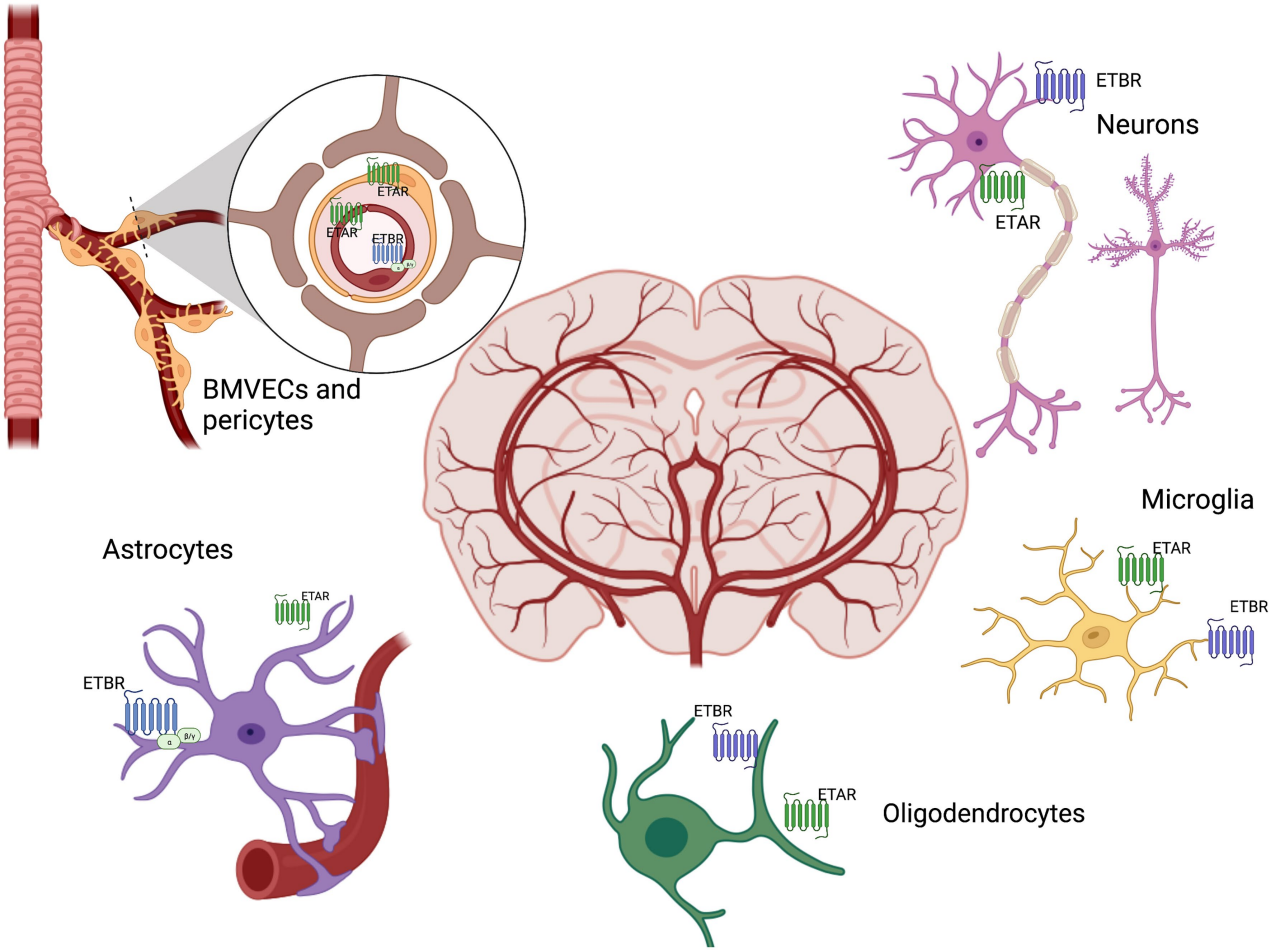
Brain ET system. ET receptors are distributed throughout the brain in all components of the neurovascular unit, including neurons, brain microvascular endothelial cells (BMVECs), pericytes, astrocytes, microglia, oligodendrocytes. ET exerts both beneficial and adverse biological actions through its receptors ETAR and ETBR in the brain.

Endothelium is an early target in diabetes resulting in cerebrovascular dysfunction due to impairment of vasoconstrictor and vasodilator responses (Ergul, 2011). The family of endothelins, consisting of three related vasoactive peptides, ET-1, ET-2, and ET-3, plays important roles in cardiovascular (patho)physiology and embryonic development. ET-1, the first isoform isolated from endothelial cells as the most potent vasoconstrictor, was later found to have proliferative, profibrotic, prooxidative and proinflammatory properties. ET peptides mediate these diverse functions *via* two distinct G protein-coupled receptor subtypes, ET_A_ and ET_B_. The ET_A_ receptor, localized mainly on vascular smooth muscle cells (VSMCs) of arteries and arterioles as well as on pericytes surrounding capillaries, is responsible for the contractile and proliferative responses to ET-1 (Davenport et al., 2016). ET_B_ receptors are predominantly found in endothelial cells and activation of this receptor subtype results in nitric oxide generation balancing ET_A_-mediated constriction in VSMCs. However, in certain beds, this receptor can promote vasoconstriction as shown in parenchymal arterioles of the brain after ischemia (Spray et al., 2017). As depicted in Figure 3, the ET system, ET peptides and receptors, is expressed in all components of the NVU (Davenport et al., 2016) but the (patho)physiology of the ET system in the brain is inadequately studied. While it was established early that ET-3/ET_B_ are the most abundant isoform and receptor in the brain (Davenport et al., 2016), the physiological significance and whether this is altered in disease states are poorly understood (Li et al., 2018).

Studies in human postmortem tissue as well as in animal models have shown that Aβ promotes pericyte-mediated capillary vasoconstriction *via* ET-1/ET_A_ signaling (Nortley et al., 2019). Additional studies showed that the degree of tissue hypoxia correlates with Aβ and ET-1 levels in dementia patients (Palmer et al., 2012, 2013). Selective astrocytic overexpression of ET_B_ receptors has been shown to promote cognitive impairment after stroke *via* exacerbated Aβ secretion from astrocytes (Hung et al., 2015). On the other hand, ET_B_ signaling in oligodendrocytes is protective by way of increasing activity-dependent myelination in the CNS (Swire et al., 2019). In this interesting study, stimulation of the receptors by IN administration of ET_B_ receptor agonist BQ3020 prevented demyelination induced by social isolation in mice. These conflicting effects of ET receptors are also reflected in other studies with pharmacological blockade or stimulation of ET receptors. While some preclinical studies reported that ET_A_-selective or dual ET receptor blockers (ERAs) improve ischemic stroke outcomes and cognitive impairment, there are also conflicting reports with selective ET_B_ agonism preventing the development of cognitive impairment (Li et al., 2018). Gulati et al. have shown in the series of studies that ET_B_ receptor agonists have potential to be therapeutic target in neurodegenerative diseases including ischemic stroke and AD (Gulati et al., 2018). Further, Briyal et al. have shown that activation of ET_B_ receptors in a type 1 diabetes model prevented the loss of cognitive functions in intracerebroventricular Aß1-40 injected animals and prevent oxidative stress (Briyal et al., 2014). On the other hand ET_A_ or dual ET_A_ receptor antagonism have shown promising neuroprotective outcomes in diabetes, ischemia reperfusion injury and Aß1 induced cognitive dysfunction (Zhang et al., 2008; Papadopoulos et al., 2010; Briyal et al., 2011; Palmer and Love, 2011; Abdelsaid et al., 2014; Singh et al., 2014, 2016).

Our group has extensively studied the role and mechanisms by which ET-1 mediates cerebrovascular dysfunction/remodeling in type 2 diabetes (Harris et al., 2008; Ergul, 2011; Abdelsaid et al., 2014, 2016; Li et al., 2018). We reported that diabetes promotes pathological cerebral angiogenesis which can be prevented and reversed by dual ETA/ETA antagonism (Abdelsaid et al., 2014). We showed that diabetes promotes neurovascular uncoupling, decreased CBF and cognitive impairment in male animals (Prakash et al., 2013a; Li et al., 2018; Ward et al., 2018b, 2019; Chandran et al., 2020). It has been shown by us and others that the ET_A_ receptor could be found on brain microvascular endothelial cells, which were previously thought to exclusively have ET_B_ receptors (Kawai et al., 1997; Abdul et al., 2018). We also reported sex differences in the regulation of ET-1 and ET_A_ expression, especially under hypoxic/diabetes-like conditions, where ET-1 levels increased to a greater extent in BMVECs of male origin (Abdul et al., 2018, 2020).

The ET system has complex physiological and pathophysiological effects. Even though studies have suggested that ERAs are beneficial in slowing down the kidney disease that frequently occurs in diabetes, water retention has been an important adverse effect that prevented these drugs from clinical use (Davenport et al., 2016; Heerspink et al., 2019). In light of the recent study, which showed IN administration of an ET_B_ receptor agonist promoted myelination, we propose that the IN administration of selective antagonists and agonists offers a novel approach to investigate the role of the ET system in the CNS and diabetes-associated cognitive impairment.

### BDNF/proBDNF

BDNF is a neurotrophin essential for synaptic function, neural plasticity, and survival that has been linked to AD and other neurodegenerative diseases affecting cognition in diabetes (Lu et al., 2013). Lower levels of BDNF have been associated with cognitive impairment (Zhen et al., 2013) and observed in stroke patients with type 2 diabetes (Chaturvedi et al., 2020). In addition to its neurotrophic effects, BDNF also has angiogenic properties (Liu et al., 2006). Mature BDNF (mBDNF) is thought to be responsible for neuroprotective effects *via* tropomyosin receptor kinase B (TrkB) mediated signaling. Whereas its precursor protein, proBDNF, and p75^NTR^ signaling have been linked to neuronal apoptosis (Benarroch, 2015). The ratio of mBDNF to proBDNF has not been well studied in diabetes, but our laboratory has shown reduced mBDNF/proBDNF ratios in both hippocampal neurons and BMVECs cultured under diabetes-mimicking conditions (Ward et al., 2018a).

Like other growth factors, BDNF is a polar protein that does not readily cross the BBB, therefore, more direct routes of administration are being investigated. Invasive administration of BDNF has been used in animal models of AD with promising results in rescuing memory deficits, synaptic density, and cell loss (Blurton-Jones et al., 2009; Nagahara et al., 2009, 2013). Emerging evidence suggests that non-invasive IN delivery is an effective delivery strategy of BDNF to the brain. IN delivery of BDNF to the brain has been successful in models of stress (Vaka et al., 2012) and ischemic stroke (Jiang et al., 2011). Others found recently that IN delivery of 42 pmol BDNF (1 μM) rescues memory performance in a model of AD (Braschi et al., 2021). The improvement in memory performance in IN BDNF-treated mice was associated with a significant decrease in CD11b immunoreactive brain microglia, further suggesting an important role for neuroinflammation in neurodegeneration (Braschi et al., 2021). BDNF is also known to affect energy balance and body weight, which may be an important consideration in diabetes; however, at low doses IN BDNF-treated mice did not show significant loss of body weight or other adverse effects (Braschi et al., 2021). Therefore, IN BDNF appears to be a promising, non-invasive intervention that improves brain bioavailability for the treatment of cognitive complications of diabetes.

## Conclusion

As the incidences of diabetes and age-related cognitive impairment increase, the urgency to identify novel preventive and therapeutic targets and strategies intensifies. In this review, we spotlight several interventions that can be repurposed for diabetes-associated VCID and call for studies investigating IN use of these pharmacological interventions.

## Author Contributions

All authors reviewed the relevant literature and wrote different sections of the review. VW wrote the sections on intranasal drug delivery and BDNF. YA wrote the sections on cerebrovascular complications of diabetes and the ET system. AE developed an outline and wrote the introduction and the sections on insulin, iron chelators, and PDE3 inhibitors. Figures were created using BioRender by the authors. All authors contributed to the article and approved the submitted version.

## Funding

This study was supported by VA Merit Review (BX000347), VA Senior Research Career Scientist Award (IK6 BX004471), NIH RF1 NS083559 (formerly R01 NS083559) and R01 NS104573 (multi-PI, SCF as co-PI) to AE; NRSA Individual Postdoctoral Fellowship to VW (F32 HL158011-01) and a South Carolina Clinical & Translational Research (SCTR) Institute (UL1TR001460) discovery grant (SCTR2201) to YA.

## Conflict of Interest

The authors declare that the research was conducted in the absence of any commercial or financial relationships that could be construed as a potential conflict of interest.

## Publisher’s Note

All claims expressed in this article are solely those of the authors and do not necessarily represent those of their affiliated organizations, or those of the publisher, the editors and the reviewers. Any product that may be evaluated in this article, or claim that may be made by its manufacturer, is not guaranteed or endorsed by the publisher.
